# Effects of Selected Egyptian Honeys on the Cellular Ultrastructure and the Gene Expression Profile of *Escherichia coli*

**DOI:** 10.1371/journal.pone.0150984

**Published:** 2016-03-08

**Authors:** Reham Wasfi, Walid F. Elkhatib, Ahmed S. Khairalla

**Affiliations:** 1 Department of Microbiology & Immunology, Faculty of Pharmacy, October University for Modern Sciences and Arts (MSA), Giza, Egypt; 2 Department of Microbiology & Immunology, Faculty of Pharmacy, Ain Shams University, Cairo, Egypt; 3 Department of Microbiology & Immunology, Faculty of Pharmacy, Beni-Suef University, Beni-Suef, Egypt; Ben-Gurion University of the Negev, ISRAEL

## Abstract

The purpose of this study was to: (i) evaluate the antibacterial activities of three Egyptian honeys collected from different floral sources (namely, citrus, clover, and marjoram) against *Escherichia coli*; (ii) investigate the effects of these honeys on bacterial ultrastructure; and (iii) assess the anti-virulence potential of these honeys, by examining their impacts on the expression of eight selected genes (involved in biofilm formation, quorum sensing, and stress survival) in the test organism. The minimum inhibitory concentration (MIC) of the honey samples against *E*. *coli* ATCC 8739 were assessed by the broth microdilution assay in the presence and absence of catalase enzyme. Impacts of the honeys on the cellular ultrastructure and the expression profiles of the selected genes of *E*. *coli* were examined using transmission electron microscopy (TEM) and quantitative real-time polymerase chain reaction (qPCR) analysis, respectively. The susceptibility tests showed promising antibacterial activities of all the tested honeys against *E*. *coli*. This was supported by the TEM observations, which revealed “ghost” cells lacking DNA, in addition to cells with increased vacuoles, and/or with irregular shrunken cytoplasm. Among the tested honeys, marjoram exhibited the highest total antibacterial activity and the highest levels of peroxide-dependent activity. The qPCR analysis showed that all honey-treated cells share a similar overall pattern of gene expression, with a trend toward reduced expression of the virulence genes of interest. Our results indicate that some varieties of the Egyptian honey have the potential to be effective inhibitor and virulence modulator of *E*. *coli* via multiple molecular targets.

## Introduction

Development and spread of antibiotic resistance is an alarming threat to effective treatment and prevention of bacterial infections in humans and animals. Solving this problem necessitates searching for natural antimicrobial alternatives [1]. Currently, more researchers are turning their attention to traditional medicines as a potential source of antimicrobial agents [2–4].

Honey is one of the oldest traditional medicines that has been highly reputed and widely used for the treatment of several human diseases for thousands of years ago [5]. This reputation has continued up to the present day, leading to the emergence of a relatively new branch of alternative medicine, called "apitherapy", which focuses on medical applications of honey and other bee products [6, 7]. Nowadays, different types of honey have been used in many countries as an alternative to pharmaceutical products for treating contaminated, infected, and burn wounds [7–10]. This is attributed to the effectiveness of these honeys in inhibiting or killing a broad spectrum of bacteria [11–13]. As a group, the Enterobacteriaceae are among the most frequently isolated bacteria in the clinical microbiology laboratory, with a high incidence of multi-drug resistance [14, 15]. As a member of this group, *E*. *coli* is particularly interesting because it has been recognized as one of the most frequently isolated bacteria in nosocomial and surgical-site infections [16, 17]. Although some studies have examined the effects of honey on bacterial structures [18–23], the majority of these studies were conducted on one type of honey, known as Manuka honey, and were mostly focused on *Pseudomonas aeruginosa* and *Staphylococcus aureus*. Therefore, more efforts are required to extend the structural studies to other honey types and other microorganisms, including *E*. *coli*.

The antimicrobial activity of honey may be attributed to several factors, including high osmolarity, acidity, in addition to the presence of hydrogen peroxide (H_2_O_2_) [24] and non-peroxide components, such as methylglyoxal [25]. In addition to exerting direct antimicrobial effects, some honey varieties have been implicated in the differential expression of a number of genes essential for bacterial survival and virulence, including those involved in stress tolerance [26], virulence factor production [27], as well as multicellular behaviors, such as biofilm formation [28], and quorum sensing [29]. To the best of our knowledge, only two published studies to date have focused on the honey-induced expression patterns in *E*. *coli* [26, 30]. In the first study, certain honeys obtained from New Zealand and Australia have been shown to modulate the expression of a large set of genes in this organism, including those involved in stress responses [26]. In the second study, different types of honey from Korean and American origins have been shown to downregulate the expression of multiple genes involved in biofilm formation, quorum sensing, and virulence in the pathogenic *E*. *coli* (O157:H7) strain [30].

Honey’s composition (and hence its antimicrobial activity) is dependent on the environmental and geographical locations from which the original nectar was collected [31]. This is attributed to natural variations in floral sources and climatic conditions at different locations [31]. Therefore, several researchers have explored the therapeutic effects of honeys obtained from diverse geographical areas worldwide [22, 32–34]. Since the ancient Egyptians used honey as a universal remedy for nearly all diseases (including bacterial infections) [5, 35–38], it was intriguing to investigate whether, and how, some of the currently available Egyptian honeys can be used effectively as antibacterial agents against *E*. *coli*, which is a predominant bacterial species in wound, urinary, and gastrointestinal tract infections [16, 17, 39–41]. Several studies have addressed different aspects of Egyptian honey varieties, including their physico-chemical properties [42–45], their chemical composition [46–51], their antibacterial and anti-biofilm activities [28, 31, 52–61], and their therapeutic usefulness [55, 62–66]. However, it is not yet known whether these anti-biofilm activities, as well as any possible anti-quorum sensing and anti-virulence activities possessed by these honeys could be attributed to alteration of bacterial gene expression. Therefore, the objectives of the present study were threefold: (i) to evaluate the direct antimicrobial activity of three of the most common Egyptian honeys against *E*. *coli in vitro*; (ii) to study the effects of these honeys on *E*. *coli* at the ultrastructural level; and (iii) to estimate the impacts of these honeys on the expressions of virulence-related genes, with focus on genes involved in biofilm formation, quorum sensing, and stress survival (in other words, to evaluate the anti-virulence potential of these honeys).

Due to financial constraints, the capacity for differential gene expression testing in the present study was restricted to a limited number of genes and not to the entire genome. Since no data are available in the literature regarding the effects of Egyptian honeys on gene expression in *E*. *coli*, selection of the genes examined was based on (i) the two published expression profiling studies, in which *E*. *coli* cells have been treated with honeys belonging to geographical and/or floral origins other than those tested in the current study [26, 30], and (ii) previous studies illustrating the genes associated with the fitness and the virulence potential of this organism [67–74]. Based on these two criteria, eight genes (namely, *yjfO* (*bsmA*), *csgA*, *ycfR* (*BhsA)*, *tnaA*, *lsrA*, *evgA*, *rpoS*, and *H-NS*) were selected for the expression analysis.

The antimicrobial sensitivity results obtained in the current study revealed the direct antibacterial activities of the tested Egyptian honeys against *E*. *coli*, which were further supported by the morphological and structural investigations. Differential gene expression results, in response to honey exposure, exhibited downregulation of several genes involved in biofilm formation, quorum sensing, and stress survival in the test organism. The ability to downregulate the expression of these genes is considered advantageous for the prophylactic and/or therapeutic use of these honeys. To the best of our knowledge, these data provide the first report that some Egyptian monofloral honeys are effective against *E*. *coli* with a dual mechanism that involves the direct growth inhibition and the downregulation of a number of fitness- and virulence-related genes.

## Materials and Methods

### Honey Samples

Three certified monofloral Egyptian honey samples of different floral origins, namely, citrus (*Citrus spp*.), Egyptian clover (*Trifolium alexandrinum*), and Marjoram (*Origanum vulgare*), were obtained from the apiary of the Bee Research Department, Plant Protection Institute, Ministry of Agriculture, Cairo, Egypt. All honey samples were packed and sealed in airtight amber glass bottles and stored at room temperature until further analysis. To evaluate the effect of osmotic pressure, artificial honey prepared according to Cooper *et al*. [75] was included in the analyses as a control.

### Free, lactonic, and total acidity of honey

Honey acidity was determined by a titrimetric method (AOAC, 1990; Official Method 962.19) [76]. Briefly, 10 g of honey were dissolved in 75 ml of distilled water and the pH was assessed by means of a digital pH meter (Hanna Instruments, Woonsocket, RI, USA). The honey solutions were then titrated with 0.05 N NaOH solution until pH 8.5 was achieved (to determine the free acidity). This was followed by an immediate back titration with 0.05 N HCl until the pH reached 8.3 (to determine the lactonic acidity). Total acidity was obtained by adding free and lactonic acidities. Results were expressed as milli-equivalent of acid per kilogram (meq kg^-1^) of honey.

### Total and non-peroxide antibacterial activity of honey

Honey samples were tested for antimicrobial activity against *E*. *coli* ATCC 8739 as the bacterial model strain, obtained from the American Type Culture Collection (Rockville, MD, USA). Using sterile 96-well microtiter plates with flat bottom (Nunc, Roskilde, Denmark), the minimum inhibitory concentrations (MICs) were determined by the broth microdilution assay, as described by Zainol, Mohd Yusoff [77], with minor modifications. All honey solutions were freshly prepared before each assay. All assays were performed in triplicate samples and were repeated three times to obtain reliable results.

To determine the total (peroxide and non-peroxide) antibacterial activity, a series of concentrations of each type of honey was prepared in Mueller Hinton broth (Oxoid, Hampshire, England) to obtain concentrations of 90% (w/v), 70%, 50%, 25%, 12.5%, 6.25%, 3.125%, 1.56%, 0.78%, and 0.39%. These honey solutions were filtered through 0.45μm filters (Sartorius AG, Germany) and 180 μl of the filtrate was placed in each of the test wells of microtiter plates. The working bacterial culture was prepared by adjusting an overnight culture of the test organism to 0.5 McFarland standard and further diluting it in Mueller Hinton broth to yield a working bacterial suspension of approximately 5 x 10^6^ CFU/ml. The microtiter wells were then inoculated with 20 μl of the working bacterial suspension to achieve a final test concentration of bacteria of approximately 5 x 10^5^ CFU/ml in each well. The plates were incubated at 37°C for 24 h under a constant agitation speed of 120 revolutions per minute (rpm) in a shaker incubator (Stuart Scientific, UK). Positive (growth) controls (consisting of inoculated broth), and negative (sterility) controls (containing uninoculated broth and honey) were included. Wells with the lowest concentration of honey that showed no visible growth were regarded as the MIC [23]. In addition to their visual determination, MICs were confirmed by measuring the absorbance at 590 nm using a microtiter plate reader (Stat-Fax 2100, Awareness Company USA).

The non-peroxide antibacterial activity of the honey samples was evaluated as described above, with the exception that catalase (Sigma, C1345; at a final concentration of 1% w/v) was added to each honey dilution to remove its content of H_2_O_2_. Positive (growth) control wells (consisting of inoculated broth and catalase), and negative (sterility) control wells (containing uninoculated broth, the corresponding honey, and catalase) were processed along with each honey sample subjected to the assay.

### Effect of honey on bacterial structure using Transmission Electron Microscope

The effect of honey on the structure of *E*. *coli* ATCC 8739 was investigated using transmission electron microscope (TEM), as described by Henriques, Jenkins [20] with slight modifications. The buffer used in cell preparation was 0.05 mM sodium phosphate buffer (pH 7.2), and cells were incubated at 37°C for 3 h without or with honey (at 1× MIC), or in buffer containing artificial honey solution (at 1× MIC) to determine the effect of sugars in honey on the cell structure. Subsequent cell fixation and processing steps were performed according to the method of Lemar, Turner [78], but cells were embedded in Epon 812 resin, not Spurr. Stained sections were examined with JEM 100 CX transmission electron microscope (JEOL, Japan) operated at 80 kV. Typically, at least three micrographs for each honey were captured at 10000 magnification power, and cells were observed for structural alterations.

### RNA isolation and cDNA synthesis

A volume of 20 ml of each of the tested honeys was prepared at sub-inhibitory concentrations (0.3× MIC) in Lauria Bertani (LB) broth medium and filter sterilized. An overnight culture of *E*. *coli* ATCC 8739 was diluted 1:100 and cells were grown in LB broth to mid-exponential phase (OD_600_ = 0.4–0.6). Aliquots of this culture (500 μl) were used to inoculate the honey solutions, which were then incubated with shaking at 37°C for 3 h. At the end of incubation, samples were washed twice with cold phosphate-buffered saline, and total RNA was isolated using Tri^®^-reagent (Sigma-Aldrich; St. Louis, MO USA) according to the recommendations of the manufacturer. Purified RNA was further treated with RNase-free DNase I (Fermentas; Grand Island, NY, USA) to eliminate DNA contamination. The concentration and purity of the isolated total RNA was determined by the ND-1000 spectrophotometer (NanoDrop Technology; Wilmington, DE, USA), and the integrity of the RNA samples was verified by agarose-gel electrophoresis. Finally, 1 μg of total RNA from each sample was reverse-transcribed to cDNA using the Maxima^®^ First Strand cDNA Synthesis Kit (Thermo Fisher Scientific, USA) as per the manufacturer's instructions. In parallel, RNA was isolated from both untreated bacterial cells and artificial honey-treated ones, and subjected to cDNA synthesis, using the same procedures.

### Quantitative real-time polymerase chain reaction (qPCR) analysis

The effects of honeys (including an artificial honey solution as an osmolarity control) on the expression levels of eight target genes [*yjfO* (*bsmA*), *csgA*, *ycfR* (*BhsA*), *tnaA*, *lsrA*, *evgA*, *rpoS*, and *H-NS*] involved in biofilm formation, quorum sensing, and stress survival in the tested organism, were examined using qPCR. The complete genome sequence of *E*. *coli* ATCC 8739 was obtained from the NCBI database (GenBank accession no. CP000946.1) and used as the base for primer design. Primers for the qPCR used in the current study (Table 1) were designed using Allele ID 6 software (PremierBiosoft, USA) and synthesized by Eurofins MWG Operon (Ebersberg, Germany). PCR reactions were carried out using a Stratagene Mx3005P thermal cycler (Agilent Technologies; Santa Clara, CA, USA) and the Maxima SYBR Green qPCR Master Mix kit (Fermentas, USA). All reactions (25 μl) were performed using three technical replicates, with 200 ng cDNA and 300 nM primers per reaction. The PCR cycling conditions were as follows: one cycle with 95°C for 5 min (hot start); then 40 cycles of denaturation at 95°C for 30 s, annealing and fluorescent data collection at 53–56°C (depending on primers used) for 30 s, and extension at 72°C for 30 s. On completion of the reaction, a dissociation curve was generated to verify that a single product was amplified. In all qPCR runs, negative controls (lacking the template or the reverse transcription step) were run in parallel. The *16S rRNA* and the *ftsA* genes were selected as internal controls, and the stability of their expression among honey samples was assessed using the BestKeeper tool [79]. The relative mRNA levels of genes of interest were determined and normalized to the expression of the housekeeping genes using the GenEX software (Multi D Analysis, Sweden). The qPCR data were expressed as the changes in expression levels of genes in honey-treated *E*. *coli* ATCC 8739 as compared to their levels in the untreated bacteria.

**Table 1:** Primers used for the quantitative real-time PCR analysis in this study.

Target gene^*a*^	Forward primer^*b*^	Reverse primer^*b*^	T_a_* (°C)	Amplicon size (bp)	Reference
***yjfO* (*bsmA*)**	CGCCAGTAACGGACCATC	GTGCTTACGCTACCTATTCG	53	76	This study
***csgA***	ATGGCGGCGGTAATGGTG	GTTGACGGAGGAGTTAGATGC	56	191	This study
***ycfR* (*BhsA*)**	CGAAGTTCAGTCAACGCCAGAAG	TCCAGCGATCCCAGATTTGTCC	54	81	This study
***tnaA***	CTGGATAGCGAAGATGTG	CGGAATGGTGTATTGATAAC	54	174	This study
***lsrA***	TACTCATAACCTTCGTGGATTCTG	TACTTGCGGCGAGGCTTC	55	178	This study
***evgA***	TAGCGGAGACGATAATAATAATTC	GTTGACTGAAGGCGGAAG	53	155	This study
***rpoS***	CTCAACATACGCAACCTG	GTCATCAACTGGCTTATCC	54	199	This study
***H-NS***	CCGAACGAACTGCTGAATAG	TTACCTTGCTCATCCATTGC	54	170	This study
***ftsA***	GAAGAAGTGACGCAAGAAGATG	ACGCCCGAAAGTCCTACC	55	152	This study
***16S rRNA***	CACACTGGAACTGAGACAC	CTTCTTCTGCGGGTAACG	55	189	Wu *et al*.[102]

^*a*^ *yjfO (bsmA)*, encoding a biofilm stress and motility protein A; *csgA*, curlin major subunit; *ycfR (BhsA)*, encoding a biofilm regulator and a multiple stress resistance protein; *tnaA*, gene specifically involved in quorum sensing (encoding the tryptophanase that converts tryptophan to indole); *lsrA*, autoinducer-2 (AI-2) importer gene, which is involved in quorum sensing; *evgA*, a master transcriptional regulator and part of the two-component signal transduction system EvgS/EvgA; *rpoS*, a global stress regulator that controls the expression of various virulence genes in *E*. *coli* at the onset of stress conditions; *H-NS*, encoding a global DNA-binding protein that functions as a pleiotropic regulator of gene expression; *ftsA*, essential cell division gene in *E*. *coli*; *16S rRNA*, 16s ribosomal RNA gene sequence.

^*b*^ All primer sequences are given in the 5' to 3' direction.

*T_a_: annealing temperature.

### Statistical analysis

All statistical analyses were carried out using the GraphPad Prism (version 5; GraphPad Software Inc.; La Jolla, CA, USA). All quantitative data obtained by qPCR were presented as mean values ± standard deviation (SD). To determine significant differences between control and treated samples, the one-way ANOVA with Bonferroni’s multiple-comparison test was performed. A difference with a *P* value of <0.05 was considered to be statistically significant.

## Results

### Acidity of honey

Among the analyzed honey varieties, citrus honey showed the highest total acidity (26.5 ± 0.47 meq/kg), followed by clover (23 ± 0.19 meq/kg), and marjoram (22 ± 0.68 meq/kg). The pH values of the honeys ranged from 3.98 to 4.33 (mean value = 4.16), which increased in the following order: clover honey (pH 3.98) < citrus honey (pH 4.03) < marjoram honey (pH 4.33) (Table 2). Interestingly, when the lactonic acidity was calculated, the three honeys showed comparable levels of this acidity, in the range of 10–11 meq/kg.

**Table 2:** Acidity of the tested honeys (expressed as the mean of triplicate samples ± standard deviation).

Honey variety	pH	Free acidity^*a*^	Lactonic acidity^*a*^	Total acidity^*a*^
Citrus	4.03 ± 0.02	16.5 ± 0.54	10 ± 0.57	26.5 ± 0.47
Clover	3.98 ± 0.04	13 ± 0.76	10± 0.67	23 ± 0.19
Marjoram	4.33 ± 0.03	11 ± 0.626	11 ± 0.29	22 ± 0.68

^*a*^ meq/kg

### Antimicrobial activities

The MIC values of the three tested Egyptian honeys against *E*. *coli* ATCC 8739 are shown in Table 3. Marjoram honey was recorded as the most potent honey against the test organism, in which a dilution of 25% (w/v) was required to inhibit the bacterial growth. This was followed by clover and citrus honeys, which both showed MIC values of 50% (w/v). In the case of artificial honey, a concentration of 70% (w/v) was required to inhibit the growth of the tested organism.

**Table 3:** Susceptibility of *E*. *coli* ATCC 8739 to the tested honeys. MICs of citrus, clover, marjoram, and artificial honey on *E*. *coli* ATCC 8739 were assessed by the broth microdilution assay in the presence and absence of catalase enzyme (at a final concentration of 1% w/v), and the values were expressed as % (w/v) of undiluted honey.

Honey variety	MIC % (w/v)	
	In absence of catalase	In presence of catalase
Citrus	50	70
Clover	50	70
Marjoram	25	70
Artificial honey	70	-

Where no value is given, no test was done.

To examine the influence of H_2_O_2_ on the antimicrobial activity of the tested honeys, samples were pre-treated with catalase prior to the incubation with *E*. *coli* ATCC 8739, which was followed by evaluation of their MIC values (using the broth microdilution assay). The removal of H_2_O_2_ from the three tested honeys reduced their antimicrobial activity against *E*. *coli* ATCC 8739 with different degrees. The highest reduction in antimicrobial activity was observed in the case of marjoram honey, in which the removal of H_2_O_2_ caused an approximate three-fold increase in the MIC against *E*. *coli* ATCC 8739 (from 25 to 70% w/v) (Table 3).

### Transmission electron microscopy

Honey-treated bacterial cells were examined by TEM, and the images were compared with those from the untreated control to identify structural changes, such as altered shape, modified surface layers, the presence of electron dense material, and cellular debris.

**Fig 1:**
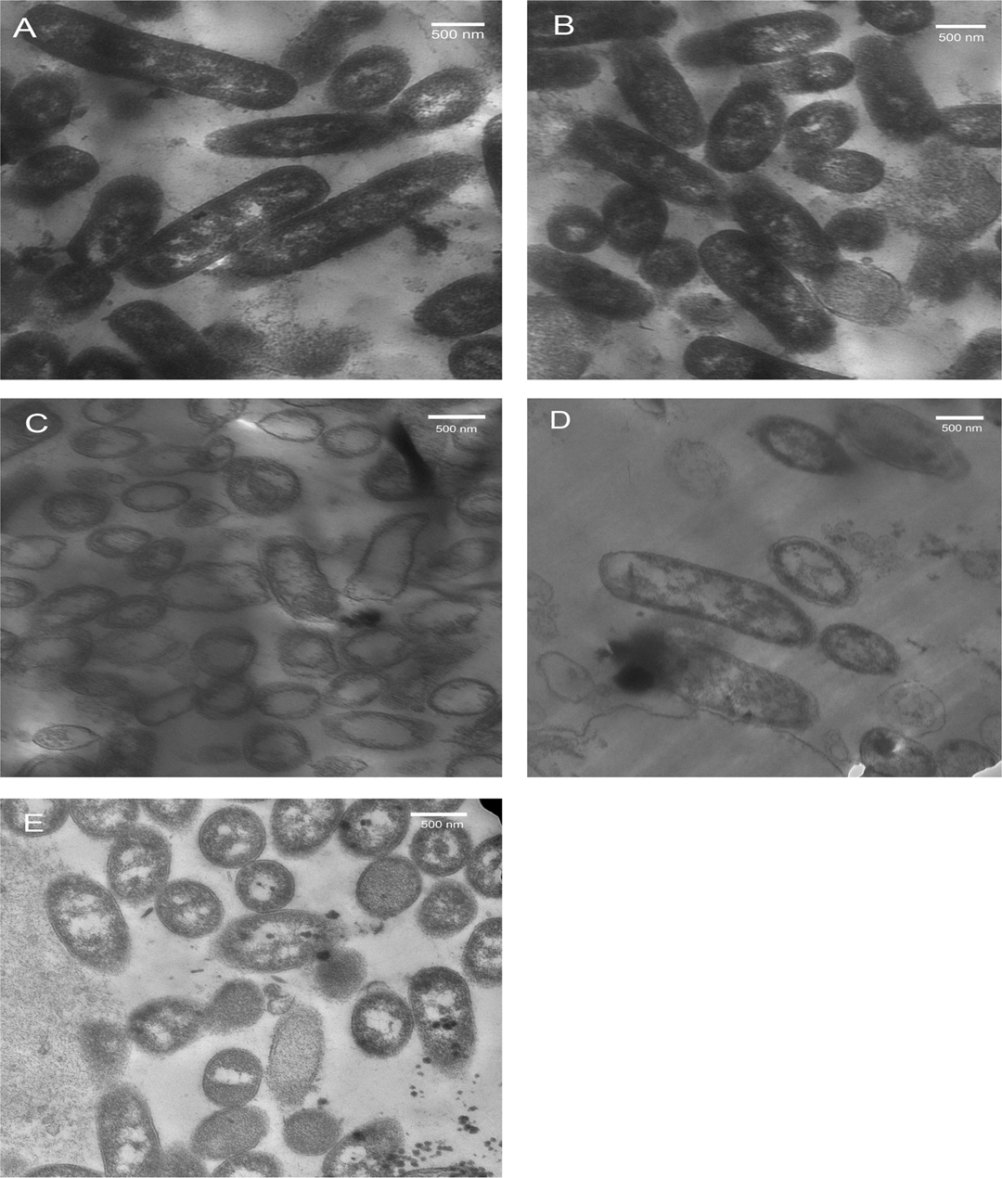
Representative TEM micrographs showing the structural changes in *E*. *coli* ATCC 8739 induced by honey treatment. Log-phase *E*. *coli* cells were treated with 1× MIC concentrations of honey for 3 h at 37°C. Cells were harvested, glutaraldehyde-fixed, embedded in Epon 812 resin, examined using a TEM (at 10,000 × magnification) and photographed (see Materials and Methods). The images represent *E*. *coli* cells: (A) of untreated control, (B) after treatment with artificial honey, (C) after treatment with citrus honey, (D) after treatment with clover honey, and (E) after treatment with marjoram honey. Scale bar = 500 nm (and is the same for A-E).

Control images of untreated *E*. *coli* ATCC 8739 cells can be seen in Fig 1A, in which the cells exhibited a uniformly dense and homogeneous microstructure, indicating that the cells were healthy.

TEM analysis revealed that no obvious morphological or structural differences exist between the bacterial cells that have been treated for 3 h with artificial honey (Fig 1B) and the untreated control (Fig 1A). In contrast, three-hour exposure to citrus honey resulted in marked changes in the bacterial cell morphology (Fig 1C). These changes included the appearance of extensively damaged cells with irregular shapes that contain an empty central zone previously occupied by the nucleoid, giving them their name, the "ghost cells" (Fig 1C).

In the case of cells treated for 3 h with clover or marjoram honey, the cells showed less electron dense materials with shrinkage of cytoplasm and vacuole formation (Fig 1D and 1E). The extent of cytoplasmic shrinkage (or disapperance) was greater in clover treated cells than marjoram treated ones, while the internal vacuolization was more extensive in marjoram treated cells (Fig 1D and 1E).

From the morphological aspect, the membranes of bacterial cells treated with citrus honey (Fig 1C) or clover honey (Fig 1D) were somewhat irregular in outline compared to untreated cells. When the lengths of bacterial cells were compared, citrus-or marjoram-treated cells (Fig 1C and 1E) were shorter than the control ones, with an average reduction of 35.8% (*P* = 0.013) and 47.8% (*P* = 0.021), respectively. On the other hand, only 5.6% reduction in length (*P* = 0.043) was observed in cells treated with clover honey as compared to the control.

### qPCR analyses

In the present study, qPCR was used to evaluate and compare the impacts of exposure of *E*. *coli* ATCC 8739 cells to three Egyptian honeys (at 0.3× MIC; for 3 h) on the expression of eight genes that have been previously shown to be involved in the fitness and virulence of the microorganism. The analysis also evaluated the effect of exposure of bacterial cells to an artificial honey solution, as an osmolarity control. The selected genes included 3 genes involved in biofilm formation [*yjfO* (*bsmA*), *csgA*, and *ycfR* (*BhsA*)], 2 genes involved in quorum sensing (*tnaA and lsrA*), and 3 genes associated with stress survival (*evgA*, *rpoS*, and *H-NS*).

**Fig 2:**
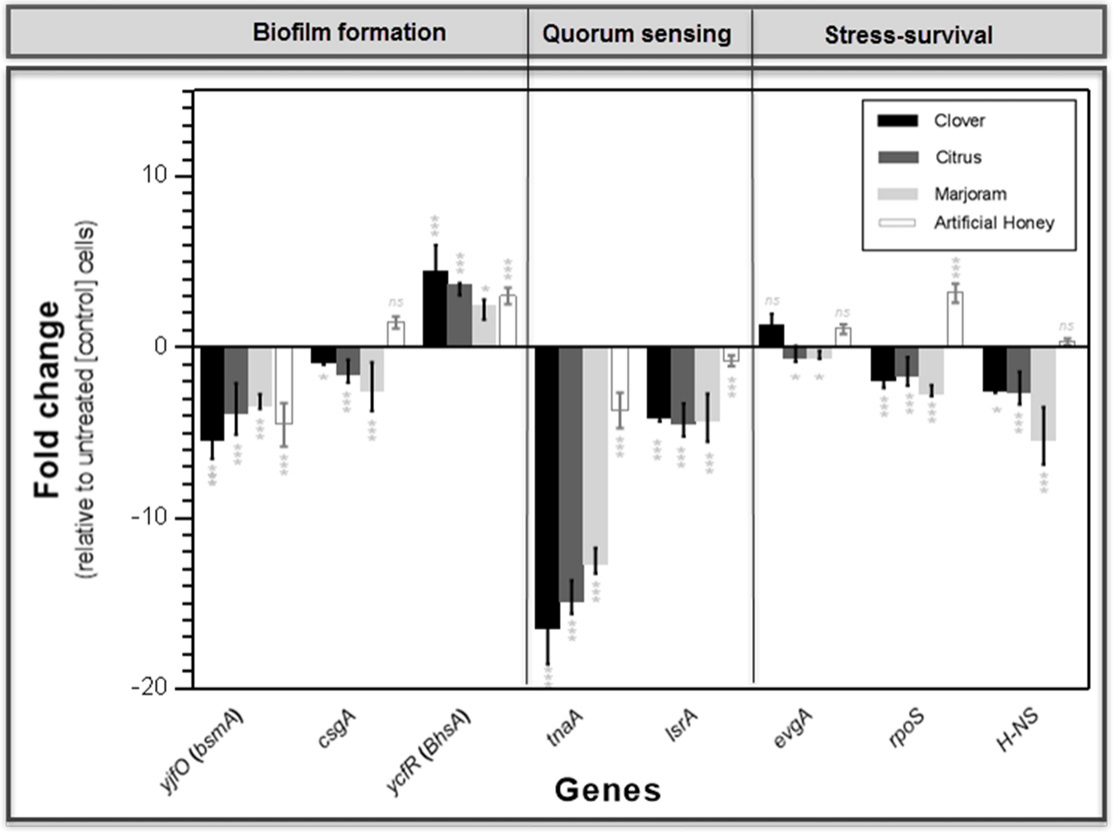
Alterations in gene expression profiles associated with exposure of *E*. *coli* ATCC 8739 to the tested honeys as determined by qPCR. Log-phase *E*. *coli* ATCC 8739 cells were exposed for 3 h to sub inhibitory concentrations (0.3× MIC) of clover, citrus, marjoram, or artificial honey. Following cell harvest, total RNA was isolated, reverse-transcribed to cDNA, and the expression levels of eight target genes (involved in biofilm formation, quorum sensing, and stress survival) in the test organism were examined using qPCR (see Materials and Methods). Experiments were run with three technical replicates of each. Mean values of fold changes (± SD) are shown in relation to untreated (control) *E*. *coli* ATCC 8739 cells. Asterisks indicate statistically significant differences in the expression of each gene between treated samples and control, as analyzed using the one-way ANOVA with Bonferroni’s correction for multiple testing (*p ≤ 0.05; **p ≤ 0.01; ***p ≤ 0.001; *ns*, no significant difference).

As revealed by the one-way ANOVA, there was a significant overall difference (*P* < 0.05) in the expression of each of the tested genes among the different groups. The *F* and the *P* values for each individual ANOVA were as follows: *yjfO* (*bsmA*) (*F*_(4, 10)_ = 15.69, *P* = 0.0003); *csgA (F*_(4, 10)_ = 13.93, *P* = 0.0004); *ycfR* (*BhsA*) (*F*_(4, 10)_ = 5.726, *P* = 0.0116); *tnaA* (*F*_(4, 10)_ = 107.9, *P* < 0.0001); *lsrA (F*_(4, 10)_ = 26.99, *P* < 0.0001); *evgA (F*_(4, 10)_ = 9.099, *P* = 0.0023); *rpoS (F*_(4, 10)_ = 57.75, *P* < 0.0001); and *H-NS (F*_(4, 10)_ = 23.56, *P* < 0.0001). Follow-up comparisons using Bonferroni's correction were performed to determine whether differences in expression of the tested genes between the exposed and control group were significant. These results were included in Fig 2.

All genes, with the exception of only two genes [*ycfR* (*BhsA*) and *evgA*] were downregulated following exposure to all natural honeys under study (Fig 2). Although different degrees of downregulation were observed following exposure to the various honeys tested, all the downregulated genes showed less than six-fold change, except for the *tnaA* gene that was downregulated in the range of 12.5–16.2 fold (Fig 2).

In the case of *ycfR* (*BhsA*) gene, its expression was upregulated following exposure to the three tested natural honeys, while the upregulation of *evgA* was observed only in clover honey-treated cells, but not in citrus or marjoram honey-treated ones. The fold increase in expression of the *ycfR* (*BhsA*) gene was in the range of 2.2–4.19 fold, while *evgA* was upregulated by 1.09 fold following exposure to clover honey (Fig 2).

To evaluate the contribution of osmolarity and acidity on the level of expression of the target genes, this assay was also performed on cDNA synthesized from RNA extracted following exposure of the cells to an artificial honey solution. In the presence of artificial honey, downregulation in the expression of all the tested quorum-sensing genes, and upregulation in the expression of all those invovled in stress survival were observed with *E*. *coli* ATCC 8739 (Fig 2). In the case of biofilm-forming genes, exposure of *E*. *coli* ATCC 8739 cells to artificial honey caused a decreased expression of *yjfO* (*bsmA*), while it caused an increased expression of *csgA*, and *ycfR* (*BhsA*) (Fig 2).

## Discussion

**Fig 3:**
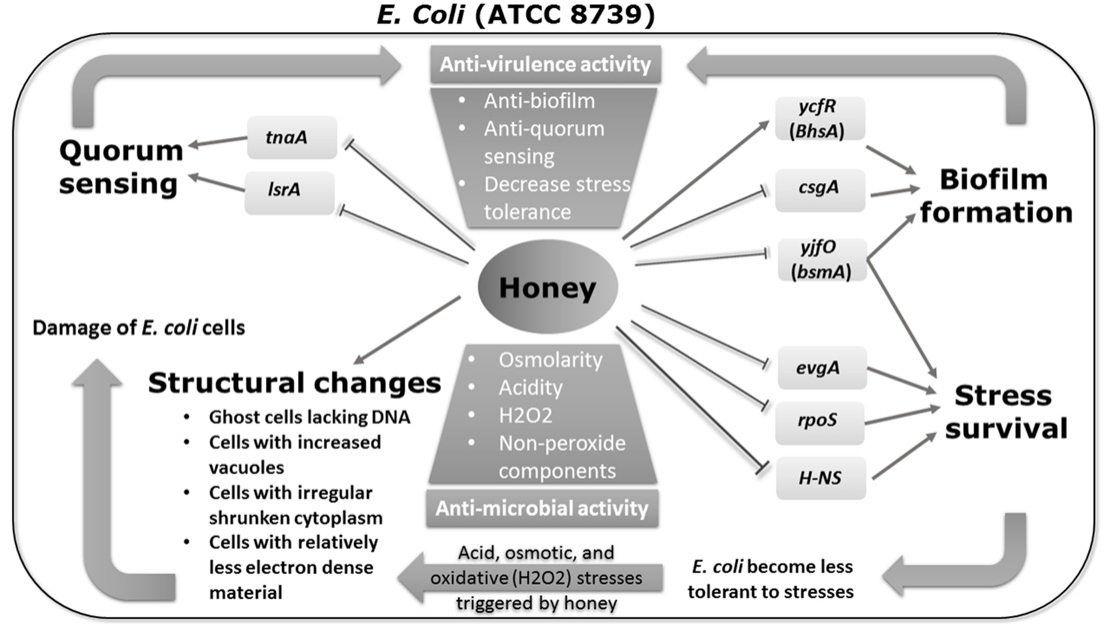
Schematic diagram summarizing the effects of the tested honeys on *E*. *coli* ATCC 8739 at both structural and molecular levels. In this figure, thin lines with pointed arrows indicate induction of gene expression or stimulation of a phenotype, while bar-headed lines indicate repression of gene expression or repression of a phenotype.

The ability of different types of honey to combat infections may be attributed to at least two complementary mechanisms. The first mechanism is attributed to their direct biocidal activity, owing to the presence of multiple factors that can damage susceptible organisms [80]. The second mechanism is mediated through their anti-virulence activity, by downregulating the expression of genes associated with virulence factor production, stress tolerance, and/or multicellular behaviors of the target organism (such as biofilm formation and quorum sensing) [80]. This latter mechanism will eventually weaken bacterial coordination, decrease their survival abilities, and interfere with their virulence mechanisms. In the current study, we investigated the antibacterial and the anti-virulence activities, in addition to the cellular changes, exerted by three Egyptian honeys from different floral sources (namely, citrus, clover, and marjoram) on *E*. *coli* ATCC 8739. To our knowledge, this is the first attempt to assess the impacts of Egyptian honey varieties on the tested organism at both structural and molecular levels. A summary of the obtained results is represented in Fig 3.

It is well known that the total antibacterial activity of honey is attributed to multiple factors, including its high osmolarity, acidity, in addition to its content of hydrogen peroxide (H_2_O_2_) and non-peroxide phytochemical components [81]. Therefore, the current study assessed these individual antibacterial factors and evaluated their relative contributions to the overall antibacterial activities of the honeys under study. To examine the effect of peroxide, the MIC values were determined in the presence and absence of catalase enzyme, which is known to break down H_2_O_2_. The possible contribution of pH and acidities (free, lactonic and total) in solutions of the tested honeys were investigated. In addition, the probable contribution of osmolarity was assessed by including an artificial honey solution in the different assays performed.

In the absence of catalase, the MIC values of the tested honeys were 1.4–2.8 fold lower than that observed with the artificial honey. This may indicate that the phytochemical components exerted a more specific growth-inhibitory activity against *E*. *coli* than the osmotic effects of sugars. In this respect, several reports have shown the high antimicrobial activity of marjoram oil against both Gram-positive and -negative bacterial strains [82, 83]. Similarly, a study conducted by Mishref and colleagues [84] has demonstrated that honey from bee colonies fed by medical herb extracts (such as marjoram) were superior in their antimicrobial activity than honey from control colonies. Our findings support and confirm those mentioned earlier, since among the honeys investigated, that derived from marjoram exhibited the highest total antibacterial activity against *E*. *coli*, as indicated by having the lowest MIC values in the absence of catalase (25% w/v). This type of honey also showed the highest levels of hydrogen peroxide-dependent activity, as the removal of its H_2_O_2_ (by catalase enzyme) caused the highest fold increase in MIC, resulting in an approximately three fold-shift.

In the current study, both citrus and clover honeys showed equal MIC values of 50% (w/v). These MICs were comparable, to some extent, with those obtained by El-Fadaly *et al*. [85], who reported equal antibacterial activities of Egyptian citrus and clover honeys against *E*. *coli*, with the MIC value of 35% (w/v). However, in contrast to our results, El-Kalyoubi, Khalaf [86] reported that Egyptian honey derived from citrus possessed higher antimicrobial activity against *E*. *coli* than that derived from clover, while the opposite order of activity was observed by Elbanna, Attalla [31]. These conflicting results may be attributed to the differences in geographical and seasonal sources, as well as harvesting, processing, and storage conditions of the honeys tested.

Turning to evaluation of acidity, the three tested honeys were acidic in nature, with pH values ranging from 3.98 to 4.33, which are likely to be low enough to inhibit the growth of many microorganisms [81]. The obtained values were in line with those of previous studies that reported a pH ranging from 3.8 to 5.2 among different Egyptian clover and citrus honeys [31, 55, 86]. Interestingly, although the total and free acidity values in marjoram honey were both less than the corresponding values of citrus or clover honeys, the antibacterial activity of the former was higher than the two latter. This observation is consistent with that of Badawy, Shafii [55], who found no correlation between free acidity of honey and its antibacterial activity against tested *E*. *coli*.

The current TEM analysis were performed on mid-exponential phase organisms because they are known to be generally more susceptible to antibacterial agents than those in the stationary phase [20]. In the current study, the TEM micrographs demonstrated the antibacterial effects of the tested honeys on *E*. *coli* ultrastructure, with the appearance of ghost cells with invisible nucleoid, cells with increased vacuoles, and/or with irregular shrunken cytoplasm, in addition to cells with relatively less electron-dense material compared to untreated controls. Several factors in the tested honeys, operating singly or simultaneously, may be involved in these alterations in the bacterial cells. The first factor may be the oxidative stress caused by H_2_O_2_ production, which is supported by the finding that the antimicrobial activities of the tested honeys are strongly peroxide-dependent. The second factor may be the acidity of honey, because this low pH is likely to: (i) inhibit the metabolism of most enzymes, (ii) alter the fatty acids responsible for cell wall synthesis, and (iii) cause protein denaturation [67]. Since inactivation of metabolic enzymes can lead to accumulation of cells with decreased size, this may explain why most of the honey-treated cells were considerably smaller than the control ones. With prolonged exposure periods (more than the 3 h applied in the current structural studies), lysis of the bacterial cells, due to alteration in membrane integrity, may eventually occur. It is noteworthy that, while the osmotic effect of sugars could be one of the involved factors, it appeared to play a minor role, if any, to the ultrastructural changes reported above. This notion is supported by the absence of obvious morphological or structural abnormalities in the artificial honey-treated cells in this study.

A number of genes have been shown to be involved in the fitness and virulence of *E*. *coli*, and thus modulating the expression of these genes can add to the effectiveness of antimicrobial therapy. Herein, eight of these genes playing important roles in biofilm formation, quorum sensing, and stress survival in *E*. *coli* were selected, and their differential gene expression profiles in response to exposure to the tested honeys were determined using qPCR.

A number of genes [including *yjfO* (*bsmA*), *csgA*, and *ycfR* (*BhsA*)] have been previously shown to be involved in the process of biofilm formation in *E*. *coli* [70, 72, 74]. The current results showed that both *yjfO* (*bsmA*) and *csgA* were downregulated after honey treatment, while *ycfR* (*BhsA*) was upregulated. This pattern of expression was the same regardless of the tested honey. The importance of this expression pattern becomes clearer when it is taken into account that *yjfO* (*bsmA*) and *csgA* have been previously characterized as biofilm-promoting genes in *E*. *coli* [70, 72], while *ycfR* (*BhsA*) has been shown to act as a biofilm repressor gene [74]. Therefore, the current findings may suggest that the honeys under study can prevent or disrupt *E*. *coli* biofilms. It has to be noted that the biological relevance of downregulating the above-mentioned genes may not be strictly limited to biofilm disruption, with a possibility to affect multiple cellular processes. In a study conducted by Weber, French [72], mutation of the *yjfO* gene in *E*. *coli* has been shown to cause alteration of cell motility, increased sensitivity to pH and oxidative stresses, and reduction of viability, rather than only affecting the biofilm formation. In another study, mutation of the *csgA* and the *lpp* (a major outer membrane lipoprotein) of an *E*. *coli* O157:H7 strain has been shown to affect HEp-2 cell invasion and motility, in addition to affecting biofilm formation [70].

A set of genes have been previously shown to play an important role in the quorum-sensing network of *E*. *coli*, such as the *tnaA and lsrA* genes [30]. The present results showed that both genes were downregulated in response to all the tested honeys. It is tempting to speculate that the tested honeys may act as quorum-sensing inhibitors, and thus may have the potential to decrease the virulence of pathogens like *E*. *coli*, by interrupting their cellular communication system. The current results are in agreement with those of Lee, Park [30], who reported downregulation of multiple genes involved in biofilm formation and quorum sensing in the pathogenic *E*. *coli* (O157:H7) strain following exposure to honey samples from Korean and American origins.

Since bacterial cells are exposed to various stressful conditions, these cells are equipped with stress survival mechanisms, being encoded in *E*. *coli* by a number of genes, including the *evgA*, *rpoS*, and *H-NS* [68, 69, 73]. Among these three tested genes, *rpoS* and *H-NS* were downregulated after exposure to all the tested honeys. The expression profile of *evgA* was quite different depending on the type of honey tested, being downregulated only in citrus or marjoram honey-treated bacteria, while it was upregulated in those treated with clover. Given the various stresses caused by honey exposure (including acid, osmotic, and oxidative stresses triggered by H_2_O_2_), it is likely that the downregulation of the above-mentioned genes will render bacterial cells less protected against stresses and damage caused by honey, which may eventually lead to loss of viability once the damage is beyond repair (Fig 3). This may, at least in part, explain the observed morphological and structural changes in honey-treated bacterial cells, and may also explain why those exposed to an artificial honey solution were still able to withstand the osmotic effect of sugars. However, our results are in contrast to those of Blair *et al*. [26], who showed marked upregulation in *rpoS*, *H-NS*, and *evgA* genes following exposure of *E*. *coli* to Manuka honey from New Zealand. This difference in expression pattern may reflect differences in the phytochemical constituents and/or differences in the antimicrobial mechanisms of the tested honeys in both studies, since Manuka honey is a non-peroxide honey [81], while the Egyptian honeys examined in our study were shown to be mainly peroxide-dependent.

Contrary to the effect of most of the tested honeys, exposure of *E*. *coli* to artificial honey resulted in upregulation of all the tested stress survival genes (*evgA*, *rpoS*, and *H-NS)* (Fig 2). This indicates that the honey-induced changes in expression of this group of genes are most probably due to specific molecules contained in these honeys and not simply due to their sugar content. In the case of the quorum-sensing genes, the three honeys under study negatively affected the expression of these genes, but the effects were more pronounced with the three honeys under study than with the artificial honey solution (Fig 2). This suggests that the anti-quorum sensing activities of the tested honeys are, at least partially, attributed to their sugar content, which is in accordance to the results of a previous study [80].

*E*. *coli* is the causative agent of the vast majority of urinary tract infections (UTIs), including those associated with catheters [87]. The biofilms frequently developed by this organism on the inner catheter lumen have always been an obstacle to effective therapy, as they facilitate bacterial adhesion to a number of biotic and abiotic surfaces, including the polymeric material forming the catheter wall [88]. It is currently known that biofilm formation in *E*. *coli* is dependent on quorum-sensing regulation [89]. Given their direct inhibitory effect on *E*. *coli*, their anti-biofilm, and their anti-quorum sensing properties, the tested honeys may represent promising materials for prevention /or treatment of UTIs, especially in catheterized patients. This may suggest their use as catheter coating, as has been demonstrated for other types of honey [90, 91]. It has to be noted that most investigations on honey-coated catheters to date were limited to those used in hemodialysis [90, 91], which may indicate the need for incorporating the tested honeys into delivery systems, to maximize their applicability as urinary catheter coating.

Biofilms are also commonly formed around implants and chronic wounds, an environment in which *E*. *coli* is one of the most consistently identified bacteria [92]. It is therefore conceivable that the tested honeys may have therapeutic potential in wound healing. In this regard, several studies have shown the effectiveness of certain honeys in treating contaminated wounds and burns when prepared in different forms, including bandages [93], dressings [94–96], nanostructured dressings [97], ointments [98, 99], and gel formulations [100, 101]. It is also conceivable that combinations of the tested honeys with antibiotics could render multidrug-resistant *E*. *coli* more susceptible to antibiotics, by interfering, at least in part, with bacterial stress tolerance and quorum-sensing mechanisms.

## Conclusion

Together, our results revealed that the tested Egyptian honeys have the potential to be effective inhibitors of *E*. *coli* and their antibacterial activities were mainly due to H_2_O_2_ generation. Marjoram honey demonstrated the highest antibacterial activity, followed by clover and citrus ones. Differential gene expression in response to honey exposure exhibited downregulation of several genes involved in biofilm formation, quorum sensing, and stress survival in *E*. *coli*. The obtained results indicate that the honeys under study may represent promising antibacterial and anti-virulence agents for treatment and modulation of infections caused by *E*. *coli*. Unlike conventional antibiotics, the tested honeys have an extra advantage, as they appear to act on multiple bacterial targets, with minimal propensity for inducing antimicrobial resistance. Future clinical evidence pertaining to the efficacy of the tested honeys in the prevention and treatment of *E*. *coli*-induced infections at various tissue/cell types might be required.
